# Two New Haplotypes of *Bartonella* sp. Isolated from *Lipoptena fortisetosa* (Diptera: Hippoboscidae) in SE Poland

**DOI:** 10.3390/insects12060485

**Published:** 2021-05-24

**Authors:** Katarzyna Bartosik, Weronika Maślanko, Alicja Buczek, Marek Asman, Joanna Witecka, Ewelina Szwaj, Paweł Szczepan Błaszkiewicz, Magdalena Świsłocka

**Affiliations:** 1 Chair and Department of Biology and Parasitology, Faculty of Health Sciences, Medical University of Lublin, Radziwiłłowska 11 St., 20-080 Lublin, Poland; alicja.buczek@umlub.pl (A.B.); szwajewelina@gmail.com (E.S.); blaszkiewicz_pawel@interia.pl (P.S.B.); 2 Department of Animal Ethology and Wildlife Management, Faculty of Animal Sciences and Bioeconomy, University of Life Sciences in Lublin, Akademicka 13 St., 20-950 Lublin, Poland; 3 Department of Parasitology, Faculty of Pharmaceutical Sciences in Sosnowiec, Medical University of Silesia, Jedności 8 St., 41-200 Sosnowiec, Poland; masman@sum.edu.pl (M.A.); jwitecka@sum.edu.pl (J.W.); 4 Department of Zoology and Genetics, Faculty of Biology, University of Bialystok, Ciołkowskiego 1J St., 15-245 Białystok, Poland

**Keywords:** *Lipoptena* sp., deer keds, invasive species, *Cervus elaphus*, ectoparasites, wild cervids

## Abstract

**Simple Summary:**

*Lipoptena fortisetosa* is a hematophagous ectoparasite of game animals feeding accidentally on companion animals and humans. Since the presence of numerous pathogenic microorganisms has been described in this species, monitoring its geographic distribution is of great epidemiological importance. To the best of our knowledge, we present two new haplotypes of *Bartonella* sp. isolated from *L. fortisetosa* in south-eastern Poland and confirm the presence of this invasive species in Lublin Voivodeship since 2013.

**Abstract:**

Insects of the genus *Lipoptena* are parasitic arthropods with a broad host range. Due to the type of parasitism (hematophagy), their potential role as vectors of pathogens, i.e., *Bartonella* sp., *Anaplasma phagocytophilum*, *Rickettsia* spp., and *Borrelia burgdorferi* is considered. As the range of their occurrence has been changing dynamically in recent years and infestations of humans have increasingly been reported, these organisms are now the subject of numerous studies. Our research aimed to present the molecular characteristics of *Bartonella* sp. detected in *Lipoptena fortisetosa* parasitizing wild cervids in south-eastern Poland. Adults of *Lipoptena* spp. were collected from carcasses of roe deer and red deer between spring and autumn in 2013. The PCR method was used to detect *Bartonella* sp. in the insects. We report two new haplotypes of the *rpo*B gene of *Bartonella* sp. isolated from *L. fortisetosa* feeding on wild cervids in south-eastern Poland and the presence of this invasive ectoparasitic species in the studied area since 2013. Phylogenetic analyses of newly obtained *Bartonella* sp. haplotypes confirmed their unique position on the constructed tree and network topology. The *rpo*B gene sequences found belonging to lineage B support the view that this phylogenetic lineage represents a novel *Bartonella* species.

## 1. Introduction

Five of the 32 species of *Lipoptena* deer keds (Diptera: Hippoboscidae) widespread in the world fauna occur in Europe [1,2]. Two species, i.e., *Lipoptena cervi* (Linnaeus, 1758) and *Lipoptena fortisetosa* Maa, 1965, inhabit the central and northern parts of the continent. In recent years, progressive expansion of *L. cervi* [3,4,5,6,7] and *L. fortisetosa* [8,9,10,11,12,13,14,15] has been observed. As shown by literature data, the spread of *L. fortisetosa* species in Europe was most likely caused by its natural dispersal outside Asia, overlapping the ranges of Siberian and European roe deer during periodic climate changes, or by introduction with alien mammal species, e.g., sika deer (*Cervus nippon* Temminck, 1838) [16,17].

In Poland, *L. fortisetosa* was first found in Lower Silesia at the end of the 1980s [18]. This deer ked species was found again in 2007–2014 on red deer (*Cervus elaphus* Linnaeus, 1758) and roe deer (*Capreolus capreolus* Linnaeus, 1758) in the north [19,20], in environments in north-eastern and southern Poland, including the Polish part of the Tatra Mountains [21,22], and recently in northern and western Poland [23].

Depending on the geographical region as well as climate and ecological conditions, the level of prevalence and severity of invasion of specific ectoparasites varies significantly, e.g., in the case of *L. cervi*, it mainly depends on the host species [4,24] and exhibits seasonal differences: the highest prevalence is most often noted in autumn and winter [25]. *L. cervi* parasitize domestic and wild animals, primarily representatives of Cervidae-red deer, roe deer, and moose (*Alces alces* Linnaeus, 1758) [15,26], own observations. The *L. fortisetosa* host species have not been clearly defined, but they are probably the same animals as the hosts of *L. cervi* [13,16]. Human infestations by *Lipoptena* adults in their habitats are increasingly being reported. Their bites cause dermatitis in humans [3,27,28,29]. In animals, the parasitism of these flies induces clinical symptoms related to anemia and skin mechanical damage [30,31].

In *Lipoptena* spp., microorganisms causing human and animal diseases have been detected. These include, e.g., *Anaplasma ovis* [32], *Anaplasma phagocytophilum* [23,33], *Bartonella* sp. [23,34,35,36,37,38,39,40], *Borrelia burgdorferi* [33], *Rickettsia* spp. [23,32], *Trypanosoma* spp. [41,42], *Coxiella*-like bacteria, *Theileria luwenshuni*, and *Theileria ovis* endosymbionts [23,43]. This fact contributed to the increased interest in the potential involvement of these arthropods in maintenance of foci of zoonotic diseases. In north-eastern Poland, researchers detected in *L. cervi* sequences of *Bartonella* sp. with 99% similarity with *B*. *schoenbuchensis* [44]. The presence of *Bartonella* sp. was also noted in *L. fortisetosa* sampled from cervids and from the environment in the northern and western parts of the country [23].

Our study presents the molecular characteristics of *Bartonella* sp. detected in *L. fortisetosa* parasitizing wild cervids in south-eastern Poland.

## 2. Materials and Methods

### 2.1. Sampling

**Figure 1:**
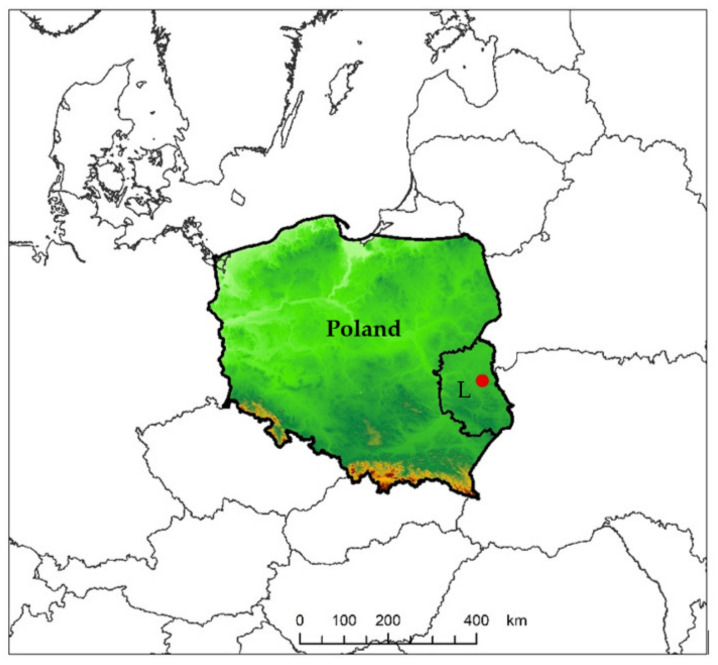
Research area in south-eastern Poland, L-Lublin Voivodeship.

Specimens of *Lipoptena* were collected in spring and autumn 2013 from carcasses of *C. capreolus* and *C. elaphus* harvested by hunters in accordance with the Act of 13 October 1995 (Hunting Law, Journal of Laws 2018, item 2033 as amended) near Polesie National Park (51°23′39″ N 23°11′4″ E) (Figure 1). These animals were culled in accordance with the Annual Hunting Plans in selected hunting circles operating in the studied macroregion, during hunting periods indicated in the Regulation of the Minister of the Environment of 16 March 2005 on the determination of hunting periods for game animals (Journal of Laws 2005, No. 48, item 459). Ectoparasites collected from the animals were placed in sterile plastic test tubes with 70% ethanol.

### 2.2. Species Identification

Identification of the species and sex of the adult insects was carried out in the laboratory using an OLYMPUS SZX16 (Olympus, Tokyo, Japan) stereoscopic microscope and the key for identification of arthropod species compiled by Borowiec [45].

### 2.3. Molecular Analysis

#### 2.3.1. DNA Extraction and Polymerase Chain Reaction

The molecular analysis included 24 specimens of *Lipoptena* spp., each blood-fed adult from a different animal host. The DNA from 15 *L. cervi* (6 females and 9 males) and 9 *L. fortisetosa* (7 females and 2 males) randomly selected for the pilot study was isolated with the ammonia method [46]. Next, its concentration was measured spectrophotometrically using a nanospectrophotometer Pearl (Implen, Germany) at a 260/280 wavelength. Then, the samples were frozen at −20 °C and stored until further analysis. The PCR method and a pair of primers (1400F and 2300R) specific to the *rpo*B gene were used to detect *Bartonella* sp. in the insects [47]. The amplification product was separated electrophoretically in 2% ethidium bromide-stained agarose gel. Then, the gel was visualized in ultraviolet light in an Omega 10 device (Ultra Lum, Claremont, CA, USA). Next, the samples were analyzed with the use of Total Lab software (TotalLab, Newcastle-Upon-Tyne, UK). The presence of an 825 base pair PCR product was treated as positive. Next, this product was isolated from the agarose gel with the use of an Agarose Out kit (EURx, Gdansk, Poland) according to the manufacture’s protocol and sequenced (Genomed, Warsaw, Poland).

#### 2.3.2. Sequencing and Phylogenetic Analysis

The resulting sequences of the *rpo*B gene for RNA polymerase beta subunit were aligned and revised manually in BioEdit v7.0.4 [48]. The obtained sequences were submitted to GenBank. To test the phylogenetic relationships among our newly obtained *rpo*B gene haplotypes and sequences downloaded from GenBank, we constructed a phylogenetic tree using a maximum-likelihood (ML) algorithm in Mega v5.05 [49] with 1000 bootstrap replicates. The GTR+I+G model of substitution was selected as the best-fitting model by the *AIC* test (Akaike Information Criterion) with jModelTest [50] for the ML tree. We also calculated and visualized the relationships among founders in our study and downloaded *rpo*B gene haplotypes from GenBank by constructing a haplotype network using the median-joining method available in Network version 10.2.0.0 (http://www.fluxus-engineering.com (accessed on 10 February 2021).

## 3. Results

Two species, i.e., *L. cervi* and *L. fortisetosa*, were identified among the *Lipoptena* adults collected from *C. elaphus* and *C. capreolus*. The preliminary analyses of the presence of *Bartonella* sp. in the deer keds involved 15 *L. cervi* specimens (3 females and 6 males from *C. elaphus* and 3 females and 3 males from *C. capreolus*) and 9 *L. fortisetosa* specimens (3 females and 2 males from *C. elaphus* and 4 females from *C. capreolus*). In total, *Bartonella* sp. were detected in 3/24 (12.5%) of the studied insects. The presence of the bacteria was shown in only 3/9 (33.3%) *L. fortisetosa* adults (2/7 of the studied females and 1/2 of studied males). No *Bartonella* sp. were detected in the *L. cervi* adults. The derived sequences of *Bartonella* sp. were submitted to the GenBank database under the accession numbers: MZ061868, MZ061869. The sequences obtained in this study share from 96.6 to 98.3% similarity with *Bartonella* sp. Honshu isolated from sika deer blood in Japan (GenBank accession no. AB703145).

**Figure 2:**
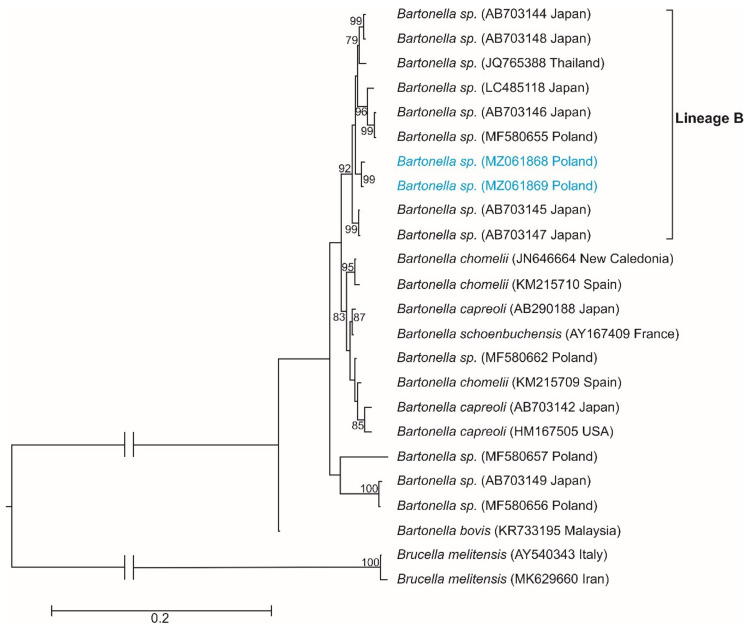
Maximum-likelihood topology computed with the GTR+I+G model of substitution evolution, representing the phylogenetic relationships among the sequences of the *rpo*B gene for RNA polymerase beta-subunit found in *Bartonella* sp. Numbers listed at the nodes represent the percent support for the node from 1000 bootstrap replicates. The ML tree has been rooted with sequences of *Brucella melitenis*, a microorganism closely related with *Bartonella* sp., as they together belong to the same order, Hyphomicrobiales. The haplotypes of *Bartonella* sp. found in this study are marked in blue. Lineage B, according to Sato et al. [51].

**Figure 3:**
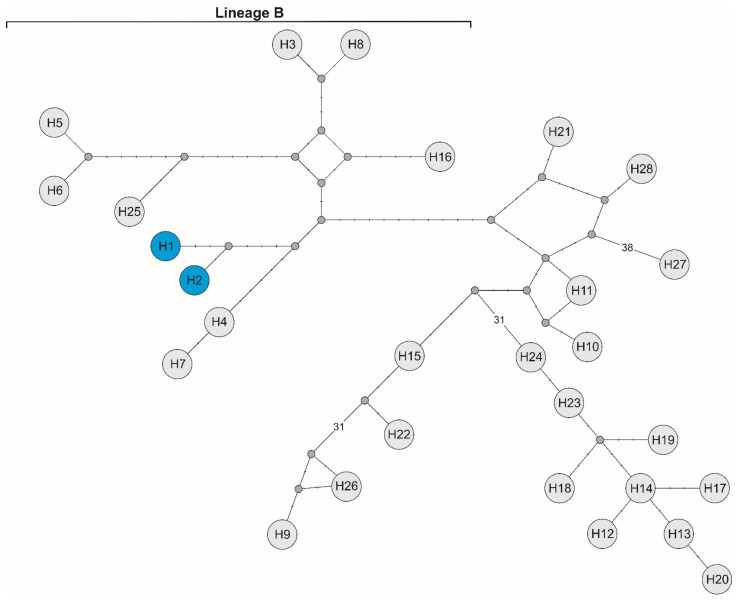
Median-joining network of *rpo*B haplotypes from Poland (H1 and H2, marked with a blue background) and haplotypes of different *Bartonella* species obtained from GenBank (H3–H28, symbols according to Table 1). Missing haplotypes are indicated by a grey dot.

The analysis of a *rpo*B gene fragment yielded two new haplotypes of *Bartonella* sp.: haplotype H1 (MZ061868) and haplotype H2 (MZ061869), as defined by three polymorphic sites, all being transitions. The maximum-likelihood phylogenetic reconstructions produced a strong topology (Figure 2). The ML tree revealed that our two *rpo*B haplotypes belong to lineage B described by Sato et al. [51]. The median-joining network based on sequences from this study and haplotypes representing different species of *Bartonella* obtained from GenBank (Table 1) suggested the presence of a distinct phylogenetic branch created by the discovered haplotypes inside lineage B. It also showed that they are grouped closely with haplotypes H4 (AB703145) and H7 (AB703147) described for new species of *Bartonella* obtained from Japanese sika deer in Japan (Figure 3).

## 4. Discussion

The zoonotic pathogen *Bartonella* sp. is a Gram-negative hemotropic bacterium, which is an etiological agent of bartonellosis. The disease usually manifests as an acute or sub-acute febrile illness in humans and animals [59]; however, a long-term symptomless infection with bacteremia in mammalian reservoir hosts (e.g., dogs and cats) was also noted [55,60,61]. The role of this bacterium as a causative agent or cofactor in endocarditis has been reported [62,63]. *Lipoptena* spp. may serve as a potential vector of this bacterium [34,35,39,40].

The prevalence of *Bartonella* sp. in *Lipoptena* is high. For instance, *Bartonella* DNA was detected in 85% of wingless adults of *L. cervi* collected from free-ranging cervids in Norway [64], and even in 94% of these deer keds collected from roe deer in France [35]. In Mazury forests (northern part of Poland), Szewczyk et al. showed the prevalence of *Bartonella* sp. in these insects at the level of 75.12% [44]. The latest data from northern and western Poland indicate the presence of *Bartonella* sp. in 49.4% of *L. fortisetosa* adults [23]. In turn, the *Bartonella* sp. infection rate in *L. fortisetosa* collected in Japan was estimated at 87.9% by real-time PCR and 51.5% in culture [40].

In this study, this bacterium was not detected in *L. cervi*. However, the absence of *Bartonella* sp. in the studied deer keds may be related to the smaller number of analyzed samples. Five sequences of *Bartonella* sp. obtained by Szewczyk et al. showed 94.4% similarity with *Bartonella* sp. from Japanese sika deer (GenBank accession no. AB703149) [44]. In turn, the two other sequences showed 99.7% similarity with *Bartonella* sp. isolated from Japanese sika deer in Wakayama Prefecture, Japan (GenBank accession no. AB703149) and with *Bartonella* sp. isolated from Japanese sika deer in Nara Prefecture, Japan (GenBank accession no. AB703146). The sequences obtained in this study showed high similarity with *Bartonella* sp. Honshu isolated from sika deer blood in Japan (GenBank accession no. AB703145) but did not show similarity with the sequences obtained by Szewczyk et al. from *L. cervi* [44].

In the maximum-likelihood (ML) algorithm based on the *rpo*B gene sequences, our two haplotypes formed a distinct branch with high bootstrap support within lineage B described by Sato et al. [51]. The ML phylogenetic analyses corroborated the result obtained from the nucleotide network and confirmed that the two haplotypes obtained in this study created a separate branch within the different species of *Bartonella*. Our newly discovered haplotypes differed by at least nine substitutions from haplotype 4 (GenBank accession no. AB703145, HonshuWD-9.3) and by at least 10 mutations from haplotype 7 (GenBank accession no. AB703147, *Bartonella* sp. HonshuWD-18.5), both described by Sato et al. [51]. Interestingly, these two GenBank haplotypes of *Bartonella* were isolated from Japanese sika deer in Japan. As shown by the network analysis, our two haplotypes and haplotypes 4 and 7 created a distinct group together, which additionally supports the view proposed by Sato et al. that lineage B represents a novel *Bartonella* species [51]. The presence of these two new *Bartonella* sp. haplotypes in *L. fortisetosa* and the haplotypes obtained by Szewczyk et al. in *L. cervi* may suggest that red deer in Poland seem to harbor the novel *Bartonella* species discovered in Japanese sika deer [44,51]. Moreover, it seems to be necessary to obtain and analyze more sequences of *Bartonella* directly from red deer blood to resolve the relationships of *Bartonella* species in deer from Japan and Poland. In turn, the role of this deer ked species as a potential vector of this bacterium needs further study.

## Figures and Tables

**Table 1:** List of species and GenBank accession numbers of their RNA polymerase beta subunit (*rpo*B) gene sequences used in the network phylogenetic analysis (Figure 3).

Symbol of Haplotype	Scientific Name	GenBank Accession Number	Sequence Source
H1	*Bartonella* sp.	MZ061868	This study
H2	*Bartonella* sp.	MZ061869	This study
H3	*Bartonella* sp.	AB703144	Sato et al. [51]
H4	*Bartonella* sp.	AB703145	Sato et al. [51]
H5	*Bartonella* sp.	AB703146	Sato et al. [51]
H6	*Bartonella* sp.	MF580655	Szewczyk et al. [44]
H7	*Bartonella* sp.	AB703147	Sato et al. [51]
H8	*Bartonella* sp.	AB703148	Sato et al. [51]
H9	*Bartonella* sp.	AB703149	Sato et al. [51]
H10	*Bartonella capreoli*	AB290188	Inoue et al. [52]
H11	*Bartonella schoenbuchensis*	AY167409	Unpublished
H12	*Bartonella bovis*	DQ356077	Unpublished
H13	*Bartonella bovis*	EF432062	Maillard et al. [53]
H14	*Bartonella bovis*	KF218218	Bai et al. [54]
H15	*Bartonella chomelii*	JN646664	Mediannikov et al. [55]
H16	*Bartonella* sp.	JQ765388	Unpublished
H17	*Bartonella bovis*	KF218217	Bai et al. [54]
H18	*Bartonella bovis*	KF218220	Bai et al. [54]
H19	*Bartonella bovis*	KF218224	Bai et al. [54]
H20	*Bartonella bovis*	KJ909808	Rudoler et al. [56]
H21	*Bartonella chomelii*	KM215709	Antequera-Gomez et al. [57]
H22	*Bartonella chomelii*	KM215710	Antequera-Gomez et al. [57]
H23	*Bartonella bovis*	KR733194	Kho et al. [58]
H24	*Bartonella bovis*	KR733195	Kho et al. [58]
H25	*Bartonella* sp.	LC485118	Sato et al. [51]
H26	*Bartonella* sp.	MF580656	Szewczyk et al. [44]
H27	*Bartonella* sp.	MF580657	Szewczyk et al. [44]
H28	*Bartonella* sp.	MF580662	Szewczyk et al. [44]

## Data Availability

The original contributions presented in the study are included in the article. Further inquiries can be directed to the corresponding authors.
